# Dual defense mechanisms from a novel marine fungus *Nigrograna chlamydospora* sp. nov. B143: antifungal and anti-aflatoxigenic properties against *Aspergillus flavus*

**DOI:** 10.3389/fmicb.2026.1801199

**Published:** 2026-04-01

**Authors:** Xiaoyun Ou, Qiaozhen Wang, Shushi Huang, Youzhi Li, Ling Yang, Yuening Luo, Shaojie Wang, Min Liang, Lixia Pan, Bin Liu, Dengfeng Yang

**Affiliations:** 1 State Key Laboratory of Non-Food Biomass Energy Technology, Guangxi Key Laboratory of Marine Natural Products and Combinatorial Biosynthesis Chemistry, Guangxi Academy of Marine Sciences, Guangxi Academy of Sciences, Nanning, China; 2 State Key Laboratory for Conservation and Utilization of Subtropical Agro-Bioresources, College of Life Science and Technology, Guangxi University, Nanning, China; 3 Department of Traditional Chinese Materia Medica, Shenyang Pharmaceutical University, Shenyang, China; 4 Institute of Applied Microbiology, College of Agriculture, Guangxi University, Nanning, China

**Keywords:** aflatoxin B1, antifungal activities, Aspergillus flavus, dual defense mechanisms, *Nigrograna* chlamydospore

## Abstract

**Introduction:**

A novel strain with strong antagonistic and anti-aflatoxigenic activity against *Aspergillus flavus* was isolated from a marine environment, highlighting its potential for biocontrol applications in food and agriculture.

**Methods:**

The strain, designated B143, was identified using a polyphasic taxonomic approach combining morphological observation and phylogenetic analysis. Its petroleum ether extract was tested for inhibitory effects on hyphal growth, spore germination, and aflatoxin B1 production in *A. flavus*. Transcriptome analysis was conducted to identify differentially expressed genes in aflatoxin and ergosterol biosynthesis pathways. Fluorescence staining was used to assess cell wall and membrane integrity, as well as oxidative stress.

**Results:**

Strain B143 was identified as a new species, *Nigrograna chlamydospora* sp. nov. Its petroleum ether extract significantly inhibited hyphal growth (70.84 ± 4.29%) and spore germination (98.23 ± 0.10%), and reduced aflatoxin B1 production by 35.88%. Transcriptome analysis showed down-regulation of key genes in aflatoxin B1 (adhA, 1.2-fold) and ergosterol (erg5, 1.6-fold) biosynthesis pathways. Fluorescence staining confirmed disruption of cell wall and membrane integrity and induction of oxidative stress in *A. flavus*.

**Discussion:**

These findings demonstrate that *N. chlamydospora* B143 exhibits strong dual inhibitory effects against *A. flavus* growth and aflatoxin production, underscoring its potential as an effective biocontrol agent for mitigating contamination in food and agricultural products.

## 1 Introduction

*Aspergillus flavus* is a common saprophytic fungus and also a pathogen for plants, animals and humans (Klich, 2007). It commonly contaminates peanuts, maize, nuts, spices, and dried fruit during storage, among them, peanuts and maize are the most severely contaminated, and directly affects their edible and economic value. As a major source of food pollution, *A. flavus* not only causes substantial crop losses and compromises food safety (Loh et al., 2010; Garcia et al., 2012; Wu et al., 2016) but also produces secondary metabolite aflatoxins, which are highly carcinogenic, mutagenic and teratogenic, posing serious risks to human health (Watanakij et al., 2020; Zhao et al., 2025). To date, more than 20 toxinic structural analogs have been reported, including aflatoxins B1, B2, G1, G2, M1, and M2 (AFB1, AFB2, AFG1, AFG2, AFM1, and AFM2) etc., among which AFB1 is the most toxic (Chen et al., 2019). The International Agency for Research on Cancer (IARC) has classified AFB1 as a Group I carcinogen (Kujawa, 1993). According to the Food and Agriculture Organization of the United Nations (FAO), approximately 25% of the world’s food are contaminated with mycotoxins each year. In Europe, aflatoxin contamination in wheat leads to annual economic losses estimated between 1.2 and 2.4 billion (Aphaiso et al., 2024). Aflatoxins exert hepatotoxic effects in humans and animals by disrupting the synthesis of DNA and RNA, which causes chromosomal abnormalities and DNA damage, thereby interfering with the synthesis of proteins and affecting cell metabolism (Alshannaq et al., 2018; Fang et al., 2020).

Physical and chemical control often lead to the loss of nutritional value and damage to the appearance of grains and food products. As a result, increasing attention has been directed toward biological control, which is more effective and environmentally-friendly (Zhang et al., 2025). In recent years, fungi and their bioactive compounds have been widely utilized to suppress the growth of *A. flavus* and the production of aflatoxins. For instance, yeasts are known to produce active volatile organic compounds (VOCs) that exhibit antagonistic effects against *A. flavus*. Notably, *Candida nivariensis* had the most effective to inhibit the hyphal growth and spore germination, and decreased aflatoxin yield (Jaibangyang et al., 2020). Hussein and Owied evaluated the impact of different extracts of *Agaricus bisporus* on the mycelial growth and aflatoxins production of *A. flavus* in maize, and found that nanoscale extract performed better than ordinary extract (Hussein and Owied, 2024). Similarly, *Alternaria alstroemeriae* and *Alternaria burnsii* demonstrated strong antagonistic activity against *A. flavus*, with the former also attenuating the biosynthesis of AFB1 and AFB2 (Ye et al., 2025). In addition, other fungi have been reported for their inhibitory effects, including non-toxin *Aspergillus* (Dorner et al., 1999; Maxwell et al., 2021), *Rhizopus oligosporus* (Kusumaningtyas et al., 2006), *Trametes versicolor* (Scarpari et al., 2016), *Trichoderma reesei* (Yue et al., 2022), *Auricularia auricula* (Yang et al., 2024), etc. It is worth noting that the search for antagonistic fungi has, to date, largely focused on non-marine environments.

Due to its unique environmental conditions, such as low temperature, high pressure, high salinity, limited light, low oxygen and oligotrophy, the ocean hosts rich diversity of marine microorganisms. Among these, marine-derived fungi play a significant role by producing diverse bioactive metabolites (Lan et al., 2023). These secondary metabolites include polyketides (Lan et al., 2023; Ningsih et al., 2023), peptides (Ghoran et al., 2023; Lee et al., 2010; Lee et al., 2011; Zhuang et al., 2011), esters (Buatong et al., 2019), terpenes (Liu et al., 2009; Cohen et al., 2011), alkaloids (Kito et al., 2009; Liu et al., 2011; Afiyatullov et al., 2012), among others. They exhibit a range of biological activities, including antifungal, antibacterial, antioxidant, anti-viral, anti-inflammation and anti-cancer properties. Fu et al. (2025) reported the biocontrol activity of the marine-derived fungus *Epicoccum nigrum* Q8–1 against *Alternaria alternata*. Notably, the authors demonstrated that the crude extract disrupted membrane integrity through ergosterol biosynthesis inhibition, induced redox imbalance and ROS accumulation, and ultimately triggered programmed cell death. Phlorotannins from the brown alga *Sargassum ilicifolium* effectively inhibited *A. flavus* growth, with SEM analysis revealing severe morphological alterations (Gholami et al., 2025). Tayel et al. (2024) demonstrated that selenium nanoparticles phycosynthesized using *Cystoseira myrica* extract, when conjugated with chitosan nanoparticles, exhibited potent antifungal activity against *A. flavus*, completely inhibiting fungal growth in fish feed after 7 days of storage. The nanocomposite outperformed fluconazole and induced severe hyphal damage within 48 h, suggesting its potential as a biosafe feed preservative.

The genus *Nigrograna* was established by de Gruyter et al. (2013) with *Nigrograna mackinnonii* as the sole species. However, Ahmed et al. (2014) reclassified this species under the genus *Biatriospora*, thereby regarding *Nigrograna* as a synonym of *Biatriospora*. Subsequently, Jaklitsch and Voglmayr (2016) described three new species, *Nigrograna fuscidula*, *Nigrograna norvegica*, and *Nigrograna obliqua*, which differed from known *Biatriospora* species, leading them to propose the new family Nigrogranaceae. Hongsanan et al. (2020) later reinstated *Nigrograna* as a distinct genus separate from *Biatriospora*. To date, 46 species endophytic fungi reside within the healthy tissues of plants of *Nigrograna* have been reported and accepted according to Index Fungorum (2025). Despite its taxonomic recognition, natural products and their biological activities from the genus *Nigrograna* remain poorly studied. Until now, only two studies have reported metabolites from *Nigrograna* species, including ergostane-type sterols, sesquiterpenes and medium-chain hydrocarbon (Shaw et al., 2015; Zhai et al., 2023). Shaw et al. (2015) identified a novel compound (3E,5E,7E)-nona-1,3,5,7-tetraene (NTE), from the endophytic fungus *Nigrograna mackinonnii* E5202H. Zhai et al. (2023) reported four new steroids, named nigergostanes A-D, and four new sesquiterpenoids, nigbisabolanes A-D, along with three known compounds, from the endophytic fungus *Nigrograna* sp. LY66. Among these, nigbisabolane B and 23R-hydroxy-(20Z,24R)-ergosta-4,6,8(14),20(22)-tetraen-3-one showed significant anti-neuroinflammatory activity. Endophytic fungi reside within the healthy tissues of plants, such as roots, stems, and leaves, inhabiting a relatively stable environment characterized by minimal temperature fluctuations, constant osmotic pressure, and abundant nutrients. In contrast, marine fungi are subjected to complex and variable environments, necessitating adaptations to high salinity, low temperatures, high pressure, and nutrient scarcity. Endophytic fungi are capable of producing antimicrobial compounds and plant growth regulators. However, their biosynthesis is often modulated by the host genotype and environmental conditions. Due to the harsh conditions of their habitats, marine fungi have evolved unique metabolic pathways. Unlike the previously reported endophytic *Nigrograna* species isolated from terrestrial plants (Shaw et al., 2015; Zhai et al., 2023), *N. chlamydospora* was isolated from marine sediments. This distinct ecological niche—characterized by high salinity, low temperature, and oligotrophic conditions—may exert selective pressure on the fungus to evolve specialized metabolic pathways, potentially leading to the biosynthesis of structurally novel secondary metabolites.

In this study, five fungal strains were isolated from marine sediment and exhibited significant antagonistic activity against *A. flavus*. These strains were identified as a new species, *Nigrograna chlamydospora* sp. nov. Among them, strain *Nigrograna chlamydospora* B143 and its crude extract most effectively inhibited the mycelial growth and conidial germination of *A. flavus*. Additionally, AFB1 production was significantly reduced when *A. flavus* was treated with the crude extract of *N. chlamydospora* B143. At present, no known marine fungi have been reported to simultaneously inhibit *A. flavus* and reduce aflatoxin production. Preliminary experiments demonstrated that the crude extract significantly inhibits mycelial growth and aflatoxin production in *A. flavus*. However, the underlying mechanism of action remained unclear. Given that some crude extracts from plants or microorganisms are often rich in terpenoids, phenolics, and other secondary metabolites known to exert antifungal effects by disrupting fungal cell wall or membrane integrity, we hypothesized that crude extract may initially target cellular structures, leading to hyphal morphological damage. To test this hypothesis and deeper insight into its mechanism of action, we first employed scanning electron microscopy (SEM) to observe ultrastructural changes in the hyphae. Concurrently, to explore the molecular targets in an unbiased manner, we conducted transcriptomic analysis to identify differentially expressed genes and the enriched biological pathways. This approach allows us to determine whether cell structure disruption is a direct effect or a secondary consequence and to screen for potential aflatoxin-related targets. These findings lay the groundwork for elucidating a novel antifungal mechanism against pathogenic fungi and offer new insights into microbial interactions. They also strengthen the theoretical foundation for controlling *A. flavus* and contribute to the development of enhanced strategies for improving food safety and security.

## 2 Material and methods

### 2.1 Fungal strains

*Aspergillus flavus* GP15-4 (Rasheed et al., 2024) was obtained from Prof. Bin Liu at Institute of Edible Fungi, Guangxi University. It was isolated from peanuts and cultured on PDA medium and incubated for 5 days at 30°C.

### 2.2 Collection, isolation, preservation, and morphological studies

Deposit sediment samples were collected from mangrove *Aegiceras corniculatum* in Beihai, Guangxi province, China. The samples were isolated at Guangxi Academy of Sciences using the dilution coated plate method (Ignatova-Ivanova et al., 2019). Single colonies were picked up and transferred onto PDA (Potato Dextrose Agar) plates, followed by incubation at 30°C for 10 d. Pure cultures were subsequently inoculated onto various media including PDA plates, CMA (Corn Meal Agar) plates, CDA (Czapek Dox Agar) plates, MT (Martin medium) plates, MEA (Malt Extract Agar) plates, and SNA (Synthetic Low Nutrient Agar) plates to observe colonial growth. The pure strains were stored at -80°C in 20% (v/v) glycerol and deposited in Marine Culture Collection of Guangxi (MCCG). Observation and photograph of microstructures were taken with Nikon Eclipse 80i microscope (Nikon Corporation, Tokyo, Japan) equipped with Nikon Digital Sight DSL1 microphotographic system (Ou et al., 2022). All microstructural measurements were conducted using Spot32 software v4.0.8 (Diagnostic Instruments, Sterling Heights, MI, United States).

### 2.3 DNA extraction, PCR amplification, and sequencing

Total genomic DNA was extracted from fresh mycelia using Chelex 100 (Walsh et al., 1991), a small amount of mycelia was collected with a sterilized toothpick and transferred into a sterilized 200 μL PCR tube containing 50 μL of 10% Chelex 100. The mycelia were rapidly ground for 30 s, incubated at 100°C for 10 min, and then centrifuged at 12,000 rpm for 1 min. Finally, the supernatant was used as DNA template for PCR amplification. Four partial gene regions were amplified in this study: The internal transcribed spacer (ITS) of nuclear ribosomal DNA, the large subunit of rDNA (LSU), the small subunit of rDNA (SSU), and a fragment of the translation elongation factor 1-alpha gene (Tef1-α). The primer pairs were used ITS1/ITS4 (White et al., 1990), LROR/LR5 (Vilgalys and Hester, 1990), NS1/NS4 (White et al., 1990) and EF1-983F/EF1-2218R (Rehner and Buckley, 2005). PCR amplification was performed according to the protocol described by Ou et al. (2022). The PCR products were sequenced from Beijing Liuhe BGI Gene Technology Co., Ltd.

### 2.4 Phylogenetic analysis

The newly obtained sequences were assembled using ContigExpress v3.0.0.0 and subsequently blasted in NCBI.^1^ The related sequences of similar species were downloaded from GenBank.^2^ All sequence datasets used to build phylogenetic trees are provided in Table 1.

**TABLE 1:** The species, isolates and GenBank accession numbers of taxa used in the phylogenetic analyses.

Taxon	Strain number	GenBank accession numbers			
		ITS	LSU	SSU	Tef1-α
*Nigrograna cangshanensis*	HKAS 83978	KY511063	KY511064	KY511065	KY511066
*Nigrograna cangshanensis*	MFLUCC 15-0253	NR_155486	NG_059778	NG_063630	-
*Nigrograna carollii*	CCF4484	LN626657	LN626682	LN626674	LN626668
***Nigrograna chlamydospora***	B141	**PV839861**	**PV839905**	**PV839924**	**PX278198**
***Nigrograna chlamydospora***	**B143**	**PV839862**	**PV839906**	**PV839925**	**PX278199**
***Nigrograna chlamydospora***	**B373**	**PV839863**	**PV839907**	**PV839926**	**PX278200**
***Nigrograna chlamydospora***	**B375**	**PV839864**	**PV839908**	**PV839927**	**PX278201**
***Nigrograna chlamydospora***	**B393**	**PV839865**	**PV839909**	**PV839928**	**PX278197**
*Nigrograna chromolaenae*	MFLUCC 17-1437	NR_168877	MT214473	-	MT235801
*Nigrograna chromolaenae*	MFLUCC 17-2079	MT310613	MT214568	MT226684	MT394628
*Nigrograna fuscidula*	MF1	KX650547	KX650547	KX650509	KX650522
*Nigrograna fuscidula*	MF3	KX650549	KX650549	-	KX650524
*Nigrograna hydei*	DUCC15293	ON911614	ON911618	-	-
*Nigrograna hydei*	MFLU 18-2073	NR_172415	MN387227	MN387225	MN389249
*Nigrograna impatientis*	MFLU 18-2072	NR_172416	MN387228	-	MN389250
*Nigrograna locuta-pollinis*	LC11691	MF939602	MF939585	-	MF939615
*Nigrograna locuta-pollinis*	CGMCC:3.24424	OR253152	OR253311	-	OR26357
*Nigrograna mackinnonii*	CBS 674.75	NR_132037	GQ387613	NG_06108	KF407986
*Nigrograna mackinnonii*	UTHSC:DI16-241	LT796847	LN907384	-	LN797087
*Nigrograna magnoliae*	MFLUCC 18-0720	MN081893	MN081892	MN081891	-
*Nigrograna magnoliae*	MFLUCC 20-0020	NR_172424	MT159622	MT159634	MT159605
*Nigrograna mycophila*	MF6	KX650554	KX650554	-	KX650527
*Nigrograna mycophila*	TDK	KX650555	KX650555	KX650510	KX650528
*Nigrograna norvegica*	TR8	KX650556	KX650556	KX650511	-
*Nigrograna norvegica*	CBS 141485	NR_147655	-	NG_063066	-
*Nigrograna obliqua*	KE	KX650558	KX650558	KX650512	KX650530
*Nigrograna obliqua*	MRP	KX650561	KX650561	-	KX650532
*Nigrograna oleae*	CGMCC 3.20243	OR853550	OR853562	OR853574	PP111471
*Nigrograna oleae*	GZAAS 21-0015	OR853551	-	OR853575	PP111472
*Nigrograna rhizophorae*	MFLU 19-1233	MN047085	MN047085	-	MN077064
*Nigrograna rubescens*	CHEM_2344	OQ400924	OQ400934	-	OQ413077
*Nigrograna rubescens*	CHEM_2177	OQ400922	OQ400932	-	OQ413078
*Nigrograna samueliana*	NFCCI-4383	MK358817	MK358812	MK358810	MK330937
*Nigrograna thymi*	MFLU 17-0497	NR_160462	NG_064431	NG_065679	KY775578
*Nigrograna yasuniana*	E8604b	HQ108005	LN626684	LN626676	LN626670
*Occultibambusa bambusae*	MFLUCC 13-0855	KU940123	KU863112	-	KU940193

Sequences newly generated in this study are in bold.

Maximum likelihood (ML) analysis was conducted using MEGA v6.06 (Tamura et al., 2013). All sequences were converted to FAST files and concatenated into combined datasets (ITS, LSU, SSU, and Tef1-α). The aligned datasets were exported in MEGA format, and the ML tree was constructed by MEGA v6.06 using the Kimura 2-parameter model. Bayesian inference (BI) analysis was performed using MrBayes v3.2.2 (Ronquist and Huelsenbeck, 2003). All sequences were converted to NEXUS files using PhyloSuite (Zhang et al., 2020), and partition homogeneity was tested with 1,000 replicates in PAUP*4 (Swofford, 2002). The best fit substitution model was selected with MrModeltest 2.31 (Nylander, 2004), and the Bayesian tree was subsequently generated using MrBayes v3.2.2. The resulting tree was visualized in FigTree v1.4.4 (Rambaut, 2016).

### 2.5 Antifungal activity of *N. chlamydospora in vitro*

Plate standoff assays were performed on PDA medium (Yang et al., 2024). Two well approximately 1 cm from the edges on both sides were punched with a hole punch (*d* = 5 mm). An inoculated *N. chlamydospora* disks were placed in one well, and the plates were incubated at 30°C for 2 d. Into the other well, 5 μL of *A. flavus* spore suspension (10^8^ CFU/mL) was inoculated, and followed by incubation at 30°C for 5 d. Colony growth was observed and documented, and the diameter of *A. flavus* was measured. The mycelial growth inhibition rate of *A. flavus* was calculated using the following formula (Owaid et al., 2017).


$$\mathrm{Inhibition}\mathrm{rate}\left(\%\right)=\frac{d_{\text{Control}}-d_{\text{Processing}}}{d_{\text{Conrol}}}\times 100\%$$

Where d_*Control*_ is the diameter of *A. flavus* colony of the control, d_*Processing*_ is the diameter of *A. flavus* colony of the treatment.

### 2.6 Preparation of the *N. chlamydospora* B143 fermentation broth and extraction of crude extracts

Disks of *N. chlamydospora* were punched with a hole punch (*d* = 5 mm) and inoculated into four 500 mL flasks each containing 300 mL of sterile potato dextrose broth (PDB) medium. The cultures were incubated at 30°C with shaking at 200 r/min for 21 d. The liquid cultures were filtered and centrifuged at 8,000 r/min for 10 min. To each bottle supernatant, an equal volume of ethyl acetate, petroleum ether, dichloromethane or diethyl ether was added, respectively, and extracted three times. The mycelia were treated with twice the volume of each solvent (ethyl acetate, petroleum ether, dichloromethane and diethyl ether), followed by ultrasonication for 60 min and extraction three times. All organic phases were concentrated using a rotary evaporator to obtain extracts, dried, and stored as crude extracts at 4°C.

### 2.7 Antagonist activities of *N. chlamydospora* B143 crude extracts against *A. flavus in vitro*

#### 2.7.1 Inhibition of mycelial growth

The crude extracts (extracted from ethyl acetate, petroleum ether, dichloromethane and diethyl ether) were diluted to 50 mg/mL using methanol. This concentration represented the maximum soluble concentration of the crude extract under conditions without precipitation or turbidity. Three wells were punched on each PDA plate, one in the center and one on each side, using a 5 mm diameter hole punch. Into each of the two side wells, 50 μL of 50 mg/mL crude extract solution was added. The center well was inoculated with 5 μL of A. flavus spore suspension (10^8^ CFU/mL). The plates were then incubated at 30°C for 5 d. In the control group, an equal volume of methanol was added to the side wells instead of the extract solution. Colony growth was observed and photographed, and the diameter of the *A. flavus* was measured. The mycelial growth inhibition rate of *A. flavus* was calculated using the formula mentioned above.

#### 2.7.2 Effects of crude extract on mycelial morphology

An *A. flavus* spore suspension was inoculated onto PDA plates amended with crude extract at a final concentration of 25 mg/mL, which was half of this maximum soluble concentration. A negative control without crude extract was also tested. The plates were incubated at 30°C for 5 d. The mycelial samples were prepared following the method described by Lopez-llorca and Hernandez (1996). Colonies were cut into 1 cm^2^ squares using a sterile scalpel and fixed with 2.5% glutaraldehyde at 4°C for 48 h. After fixation, the samples were washed with phosphate-buffered saline (PBS, pH 6.8). Mycelia were dehydrated with a graded ethanol series (50, 70, 80, 85, 90, 95, and 100%), each step for 15 min. Finally, the samples were freeze-dried, sputter-coated with gold, and observed under a scanning electron microscope (SEM, FEI Quattro S).

#### 2.7.3 Inhibition of spore germination

A 900 μL aliquot of PDB mixed with crude extracts was added into each 2 mL centrifuge tube, followed by the addition of 100 μL of an *A. flavus* spore suspension (10_8_ CFU/mL). The final concentration of crude extract was adjusted to 25 mg/mL. A negative control without crude extract was also tested. The samples were incubated at 30°C with shaking at 200 r/min for 24 h. The numbers of germinated and non-germinated spores were counted using a Nikon Eclipse 80i microscope (Nikon Corporation, Tokyo, Japan) equipped with a Nikon Digital Sight DSL1 microphotographic system. The spore germination rate and inhibition rate of spore germination were calculated according to the following formula (Li et al., 2024).


$$\mathrm{Spore}\mathrm{germination}\mathrm{rate}\left(\%\right)=\frac{\text{A}}{\text{B}}\times 100\%$$

$$\mathrm{Inhibition}\mathrm{rate}\mathrm{of}\mathrm{spore}\mathrm{germination}\left(\%\right)=\frac{\text{C-P}}{\text{C}}\times 100\%$$

Where A is the number of germinated spore, B is the total number of spores, C is the spore germination rate of the control group, P is the spore germination rate of the treatment group.

### 2.8 Transcriptome analysis

*A. flavus* spore suspension (10^8^ CFU/mL) was added to PDB containing 25 mg/mL crude extract as treatment group and without crude extract as control group, and then incubated at 30°C with shaking at 200 r/min for 5 d. After incubation, the mycelia were collected by centrifugation at 12,000 r/min for 10 min. Finally, the total RNA was extracted, and cDNA library was constructed and sequenced by Guangxi Pufei Information Technology Co., Ltd. Bioinformatics analysis included quality assessment and reference sequence alignment using HISAT software. Gene expression levels were estimated using the FPKM (fragments per kilobase of transcript sequence per millions base pairs sequenced) method (Trapnell et al., 2010). Differentially expressed genes (DEGs) were screened and analyzed by DESeq2, the genes with log2 (FoldChange) > 1 and *p*-value < 0.05 were considered. Finally, Gene Ontology (GO) enrichment analysis and Kyoto Encyclopedia of Genes and Genomes (KEGG) pathway enrichment analysis were performed (Hu et al., 2024).

### 2.9 Determination of AFB1 production by LC-MS and transcriptome analysis of genes in AFB1 biosynthetic pathway

AFB1 production was determined according to a previously described method (Xin et al., 2024). *A. flavus* was cultured in PDB medium with the addition of crude extract (treatment group) and without crude extract (negative control), under 30°C with shaking at 200 r/min for 5 d. After incubation, the broth culture was centrifuged, and an equal volume of chloroform was added to the supernatant to extract AFB1. Following phase separation, the lower layer was collected and dried using a nitrogen evaporator. Finally, the residue was dissolved with 1 mL methanol for analysis by liquid chromatography-tandem mass spectrometry (LC-MS).

### 2.10 Determination of cell membrane damage

*A. flavus* mycelia were treated with 25 mg/mL (treatment group) and without crude extract (negative control) in PDB medium at 30°C with shaking at 200 r/min for 72 h. After cultivation, the mycelia were collected and washed twice with PBS buffer (10 mM, pH 6.8). The samples were stained with 5 μg/mL propidium iodide (PI) at 37°C for 30 min, then washed and resuspended in PBS buffer (Xin et al., 2024). Finally, the mycelia were observed using fluorescence microscopy.

### 2.11 Determination of mitochondrial membrane potential

*A. flavus* mycelia were treated with 25 mg/mL crude extract (treatment group) and without crude extract (negative control) in PDB medium at 30°C with shaking at 200 r/min for 72 h. After cultivation, the mycelia were collected and washed twice with PBS buffer (10 mM, pH 6.8). MMP was assessed using a mitochondrial membrane potential assay kit with JC-1 (Solarbio, Beijing, China). Finally, the samples were observed using fluorescence microscopy.

### 2.12 Statistical analysis

All experiments were performed in triplicate. Data were analyzed with SPSS v25.0.0.0 and plotted using GraphPad Prism v8.4.0. *P* < 0.05 was considered statistically significant.

## 3 Results

### 3.1 Identification of the new species *Nigrograna chlamydospora*

**FIGURE 1:**
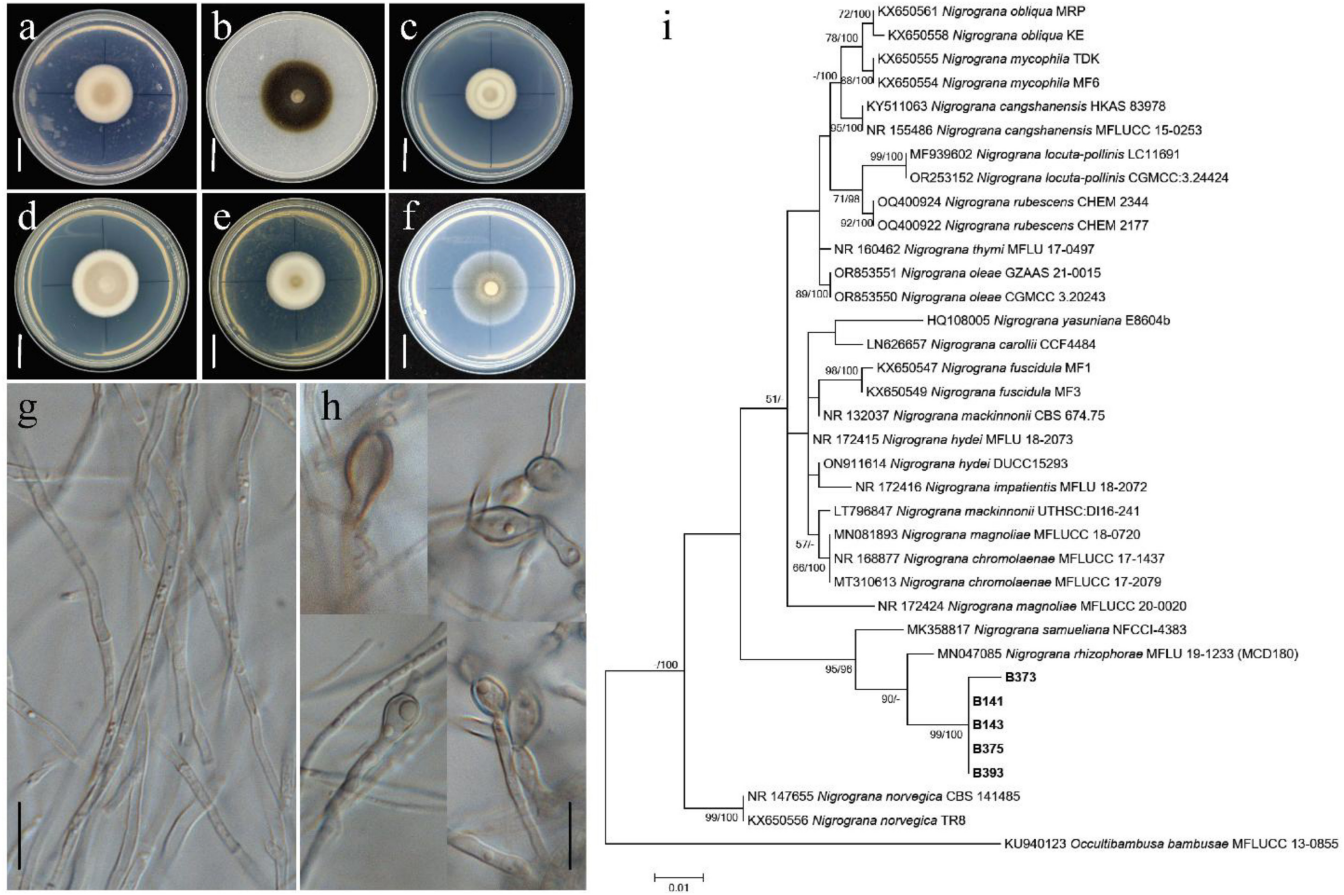
**(a–f)** Colony after 14 d at 30°C, **(a)** on PDA, **(b)** on CMA, **(c)** on CDA, **(d)** on MT, **(e)** on MEA, **(f)** on SNA. **(g)** Mycelia. **(h)** Chlamydospores. **(a-f)** From B143, **(g)** from B141, **(h)** from B141, B143 and B373. **(i)** Phylogenetic tree generated from maximum likelihood analyses based on a combined ITS, LSU, SSU and Tef1-α sequences with *Occultibambusa bambusae* MFLUCC 13-0855 as the outgroup. Maximum likelihood bootstrap support ≥ 50% (left) and Bayesian posterior probability values ≥ 95% (right) are indicated at nodes (MLBP/BIBP). Bold names represent new species. Scale bars: **(a–f)** = 20 mm, **(g–h)** = 10 μm.

*Nigrograna chlamydospora* X.Y. Ou, S.S. Huang and D.F. Yang, sp. nov. (Figure 1)

MycoBank: 860650

Etymology: according to the chlamydospores.

Holotype: CHINA, Guangxi province, Beihai city, Hepu country, sediment from *Paulownia* tree, 21°61′39″N,109°03′47″E, 12 Apr 2022, Q.Z. Wang, L. Yang, B143.

Asexual morph: Colony white on PDA (Figure 1a), 23.8 ± 0.12 mm diameter at 30°C after 14 d, aerial hyphae dense; dark olivaceous to black on CMA (Figure 1b), 34.8 ± 0.03 mm diam., aerial hyphae sparse; white on CDA (Figure 1c), 22.3 ± 0.04 mm diam., aerial hyphae dense; white on MT (Figure 1d) and MEA (Figure 1e), 28.9 ± 0.05 mm and 24.0 ± 0.08 mm diam., aerial hyphae dense; gray to light brown on SNA (Figure 1f), 32.0 ± 0.08 mm diam., aerial hyphae sparse; colony circular, raised at the center. Hyphae hyaline, septate, branched, smooth, 0.6–3.5 μm wide (Figure 1g). Chlamydospores oval to ellipsoid, hyaline, non-septate, 5.8–11.8 × 4.4–7.3 μm, the edge of cell wall is 0.4–2.0 μm thick (Figure 1h).

Phylogenetic analyses: The phylogenetic tree (Figure 1i) consisted of 36 strains with *Occultibambusa bambusae* MFLUCC 13-0855 as the outgroup, 5 strains were identified in this study. Phylogenetic analyses were based on combined sequences of ITS, LSU, SSU and Tef1-α, with aligned lengths of 396 bp from ITS, 802 bp from LSU, 782 bp from SSU and 693 bp from Tef1-α, respectively. The topology of maximum likelihood analysis was consistent with Bayesian inference analysis. The new species *Nigrograna chlamydospora* clustered with *Nigrograna rhizophorae* (MLBP = 90%, BIPP < 95%), where they formed another clade with *Nigrograna samueliana* (MLBP/BIPP = 95%/96%).

Additional specimen examined: CHINA, Guangxi province, Beihai city, Hepu country, sediment from Paulownia tree, 21°61′39″N,109°03′47″E, 12 Apr 2022, Q.Z. Wang, L. Yang, B141. CHINA, Guangxi province, Beihai city, Hepu country, sediment from Paulownia tree, 21°60′44″N, 109°03′51″E, 12 Apr 2022, Q.Z. Wang, L. Yang, B373. CHINA, Guangxi province, Beihai city, Hepu country, sediment from Paulownia tree, 21°60′44″N, 109°03′51″E, 12 Apr 2022, Q.Z. Wang, L. Yang, B375. CHINA, Guangxi province, Beihai city, Hepu country, sediment from Paulownia tree, 21°60′44″N, 109°03′51″E, 12 Apr 2022, Q.Z. Wang, L. Yang, B393.

### 3.2 Screening of *N. chlamydospora* strains for inhibition of *A. flavus* growth

**FIGURE 2:**
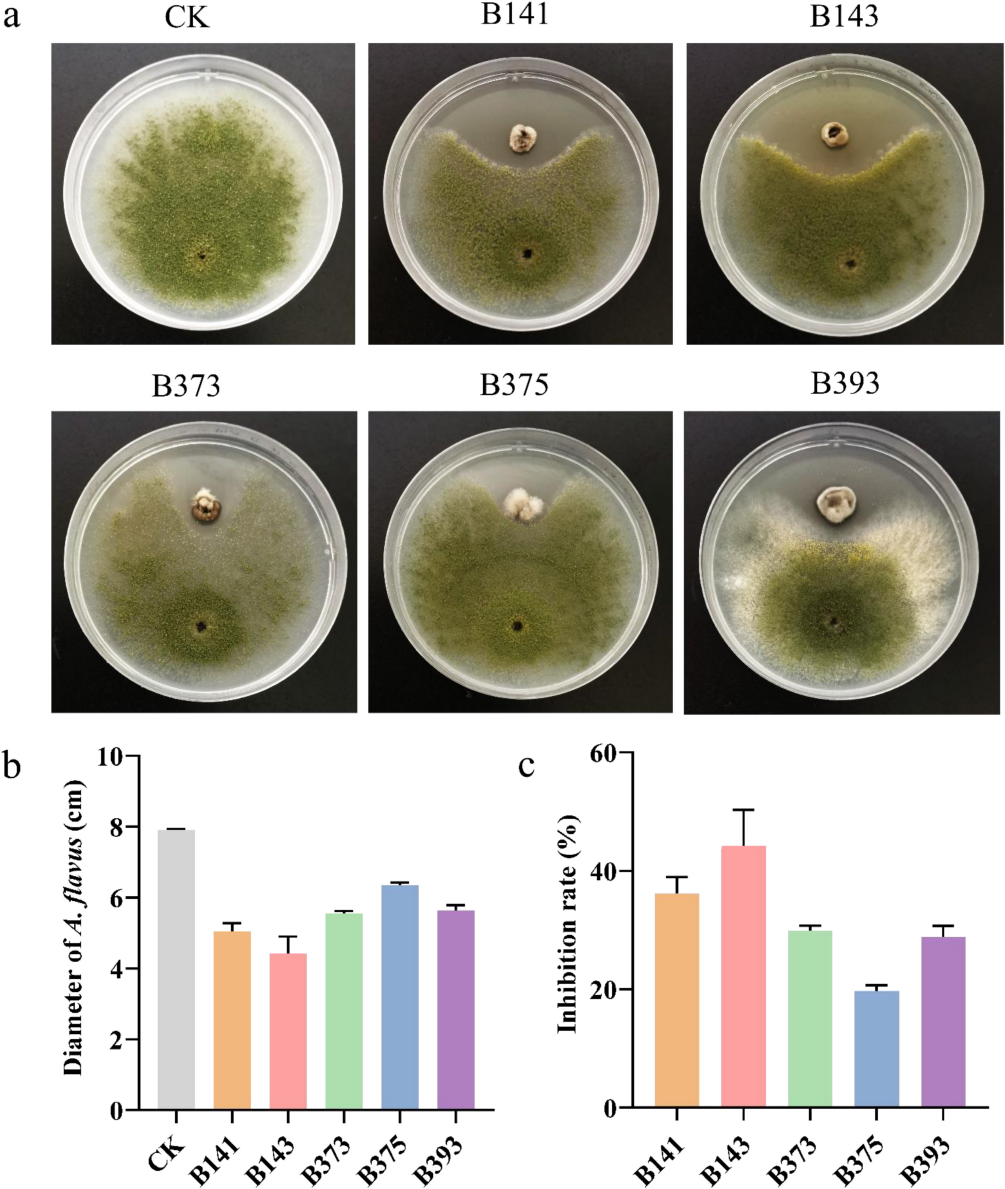
The inhibitory effect of *A. flavus* during plate standoff with *N. chlamydospora* strains. **(a)** Plate standoff between *A. flavus* and *N. chlamydospora* strains. **(b)** Colony diameter of *A. flavus* during plate standoff. **(c)** Inhibition rate of *A. flavus* during plate standoff.

The results of the plate confrontation assay between five strains of *N. chlamydospora* and *A. flavus* on PDA medium are presented in Figure 2. Compared with control, five strains of *N. chlamydospora* inhibited the mycelial growth of *A. flavus*. Among them, isolate B143 exhibited the strongest antifungal activity, while B375 showed the weakest (Figure 2a). The diameter of *A. flavus* was 7.91 ± 0.02 cm in the control group, compared to 4.42 ± 0.49 cm when confronted with B143 and 6.35 ± 0.07 with B375 (Figure 2b). The inhibition rates of the five strains with *A. flavus* were calculated as follows: 39.16 ± 2.75% (strain B141), 44.16 ± 6.14% (strain B143), 29.84 ± 0.90% (strain B373), 19.66 ± 0.98% (strain B375) and 28.78 ± 1.93% (strain B393) (Figure 2c). Isolate B143, which showed the highest inhibition rate, was selected for further study.

### 3.3 Effects of crude extracts from different polar solvents on the mycelial growth of *A. flavus*

**FIGURE 3:**
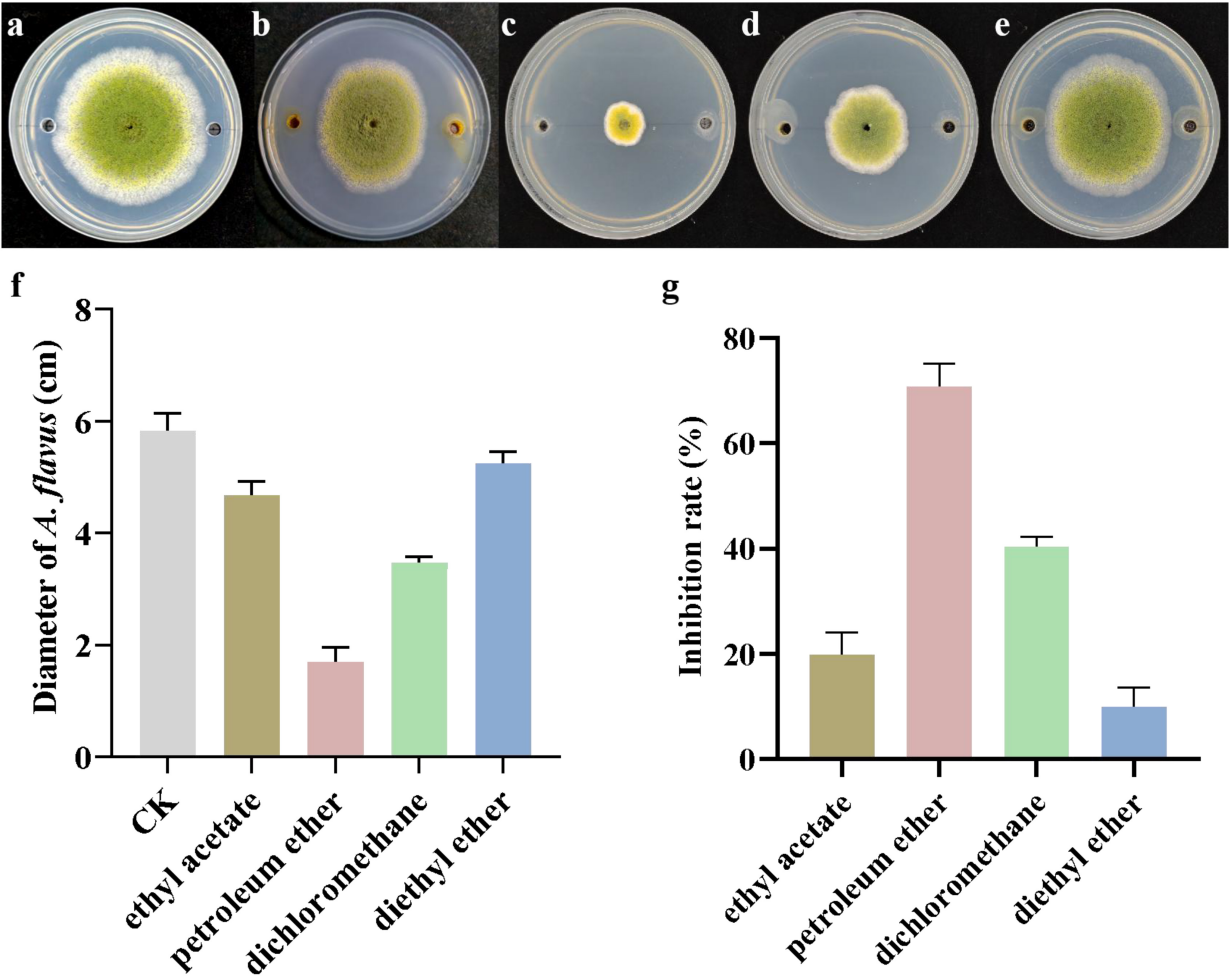
Inhibitory effects of crude extracts obtained using different polar solvents. **(a)** Control (methanol solvent). **(b)** Ethyl acetate extract. **(c)** Petroleum ether extract. **(d)** Dichloromethane extract. **(e)** Diethyl ether extract. **(f)** Colony diameter of *A. flavus*. **(g)** Inhibition rate of *A. flavus*.

The inhibitory effects of crude extracts obtained with ethyl acetate, petroleum ether, dichloromethane, and diethyl ether on the mycelial growth of *A. flavus* were assessed using the agar diffusion method. As illustrated in Figures 3a–e, all four extracts significantly suppressed fungal growth compared to the control. After treatment with the crude extracts, a significant reduction in fungal diameter was observed. Among them, the petroleum ether extract exhibited the strongest inhibitory activity, reducing the diameter of *A. flavus* to 1.70 ± 0.25 cm, in contrast to 5.83 ± 0.31 cm in the control (Figure 3f). The dichloromethane extract also showed considerable inhibition, with a diameter of 3.48 ± 0.11 cm, followed by the ethyl acetate extract (4.68 ± 0.25 cm) (Figure 3f). The diethyl ether extract had the weakest effect, yielding a diameter of 5.25 ± 0.21 cm (Figure 3f). The corresponding inhibition rates were 19.81 ± 4.24% for ethyl acetate, 70.84 ± 4.29% for petroleum ether, 40.40 ± 1.82% for dichloromethane, and 9.95 ± 3.63% for diethyl ether (Figure 3g). These results indicate that *N. chlamydospora* B143 produces metabolites capable of inhibiting the mycelial growth of *A. flavus*, with petroleum ether being the most effective solvent for extraction. Therefore, in all subsequent experiments, the crude extracts used were obtained through petroleum ether extraction.

### 3.4 Effect of crude extract on the mycelial morphology of *A. flavus*

**FIGURE 4:**
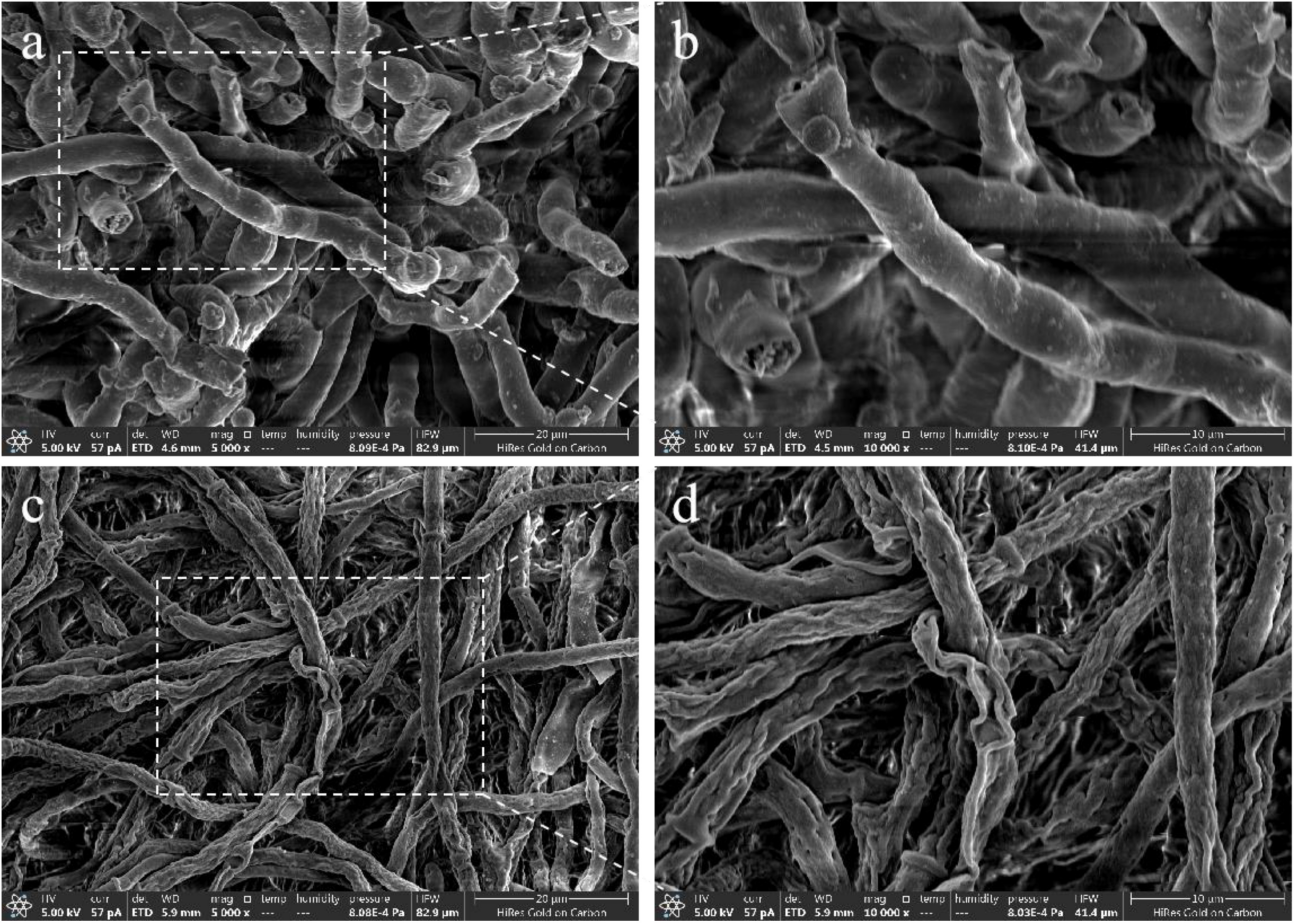
Effect of crude extract on hyphal morphology of SEM image of *A. flavus*. **(a,b)** Control (without crude extract). **(c,d)** *A. flavus* treated with crude extract from *N. chlamydospora* B143 at 25 mg/mL. **(a,c)** 5,000 × . **(b,d)** 10,000 × .

The effect of crude extract on the microstructural morphology of *A. flavus* mycelia was investigated using SEM after following incubation at 30°C for 5 d. Hyphae in the control group exhibited a regular, rounded morphology with a smooth and thick surface (Figures 4a,b). In contrast, treatment with the crude extract, resulted in pronounced morphological alterations, including distorted and flattened hyphae with rough, concave, and folded surfaces (Figures 4c,d). These observations indicated that the crude extract from *N. chlamydospora* B143 disrupts the cellular integrity of *A. flavus* mycelia.

### 3.5 Effect of crude extract on spore germination of *A. flavus*

**FIGURE 5:**
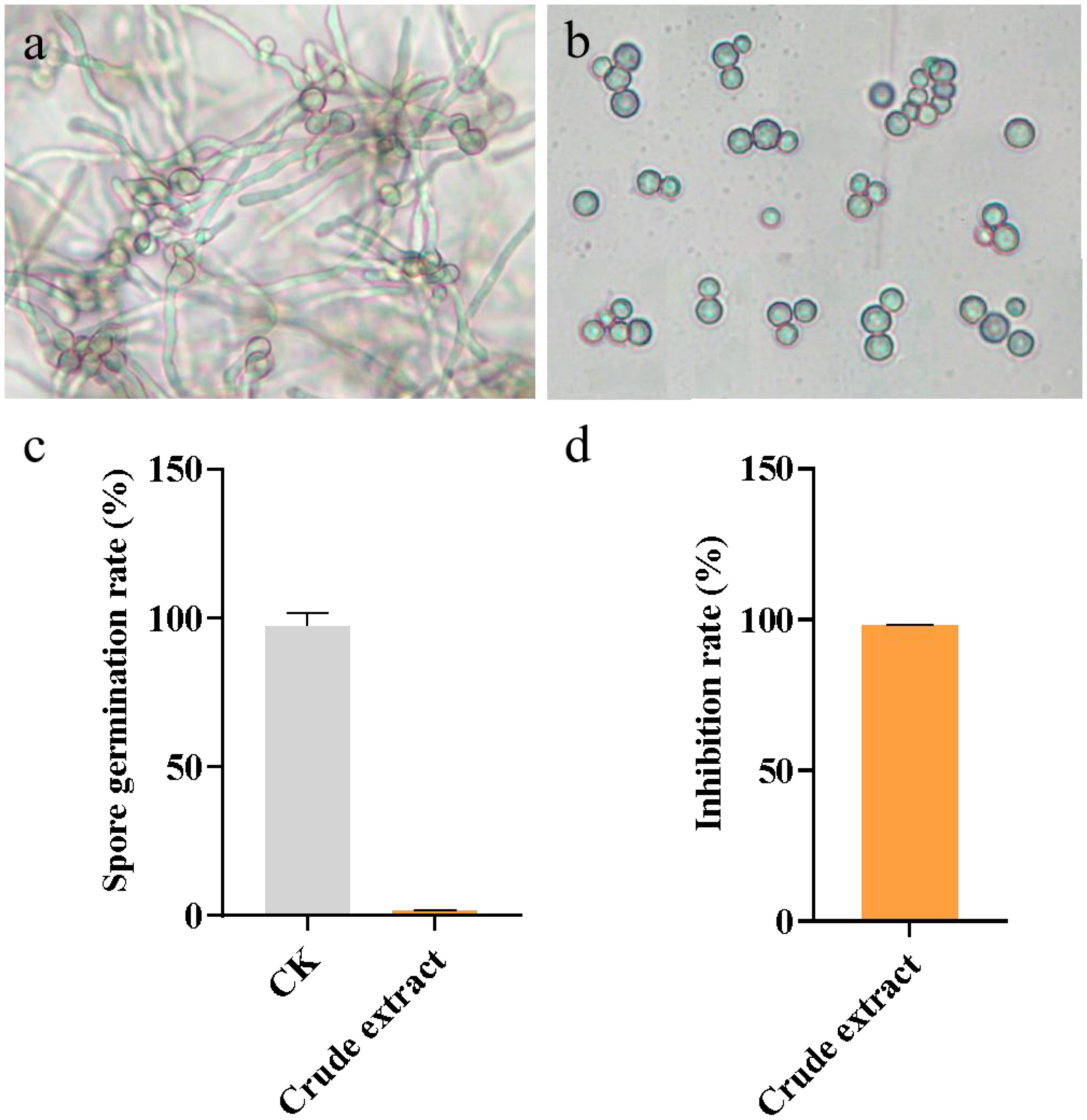
Effect of crude extract on spore germination of *A. flavus*. **(a)** Control (PDB without crude extract). **(b)** Spores of *A. flavus* treated with crude extract at 25 mg/mL. **(c)** Spore germination rate of *A. flavus*. **(d)** Inhibition rate of spore germination of *A. flavus*.

The inhibitory effect of the crude extract from *N. chlamydospora* B143 on the spore germination of *A. flavus* is shown in Figure 5. After 24 h of incubation, both the number of germinated spores and the total spore count were quantified. In the untreated control group (Figure 5a), spores germinated fully under the same conditions, achieving a germination rate of 97.44 ± 4.44% (Figures 5a,c). Exposure to the crude extract at a concentration of 25 mg/mL almost completely inhibited spore germination, with a germination rate of only 1.73 ± 0.09% (Figures 5b,c). The inhibition rate of spore germination by the crude extract reached 98.23 ± 0.10% (Figure 5d). These results indicate that the crude extract from *N. chlamydospora* B143 exhibits strong inhibitory activity against the germination of *A. flavus* spores.

### 3.6 Transcriptomic analysis of *A. flavus* treated with crude extract

**FIGURE 6:**
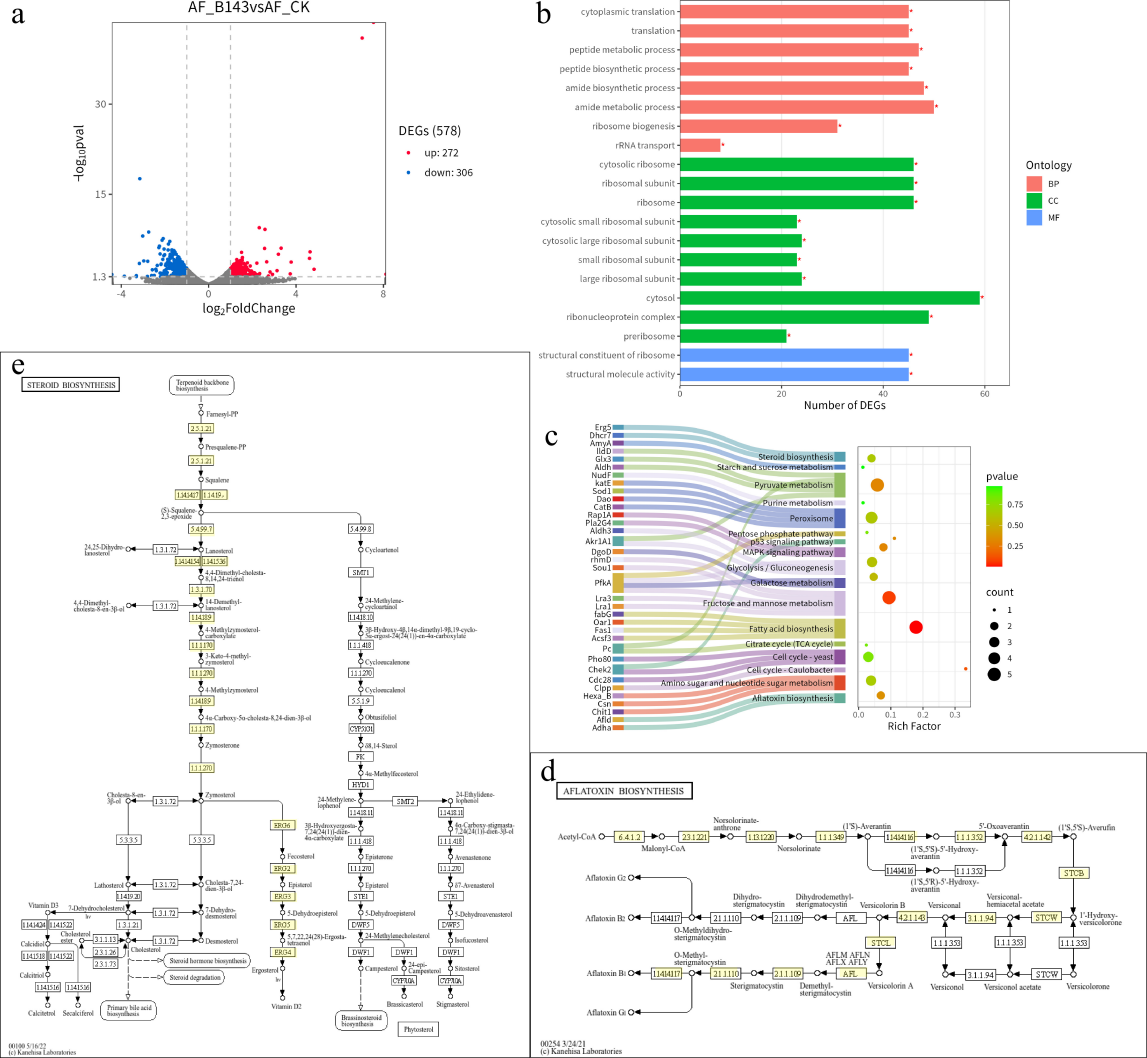
Transcriptomic analysis of *A. flavus* treated with crude extract. **(a)** Volcano plot of differentially expressed genes. Blue, red and gray points represent down-regulated, up-regulated and insignificant genes, respectively. The horizontal axis represents log2(FoldChange), and the vertical axis represents -log10 (*p*-value). **(b)** Histogram of KEGG enrichment analysis for differentially expressed genes. * indicates significantly enriched GO terms. **(c)** Sankey and dot plot representing enriched terms. **(d)** Schematic diagram of aflatoxin biosynthesis. **(e)** Schematic diagram of steroid biosynthesis. Panels **(d,e)** is from the KEGG database at https://www.genome.jp/kegg/.

To investigate the effect of crude extract on *A. flavus*, a transcriptomic analysis was conducted. Differentially expressed genes (DEGs) are presented in the volcano plot (Figure 6a). Compared with the control, 578 DEGs were identified, of which 272 were up-regulated and 306 were down-regulated. KEGG enrichment analysis indicated that the DEGs were involved in 20 pathways, among which the ribosome pathway was the most significant (Figure 6b). Ribosomes are cellular factories responsible for protein synthesis, a process requiring substantial energy. Therefore, we speculated that the crude extract of *N. chlamydospora* B143 may cause protein damage and disrupt membrane structures.

To further elucidate the effects of crude extract on the mycelial growth and aflatoxin production of *A. flavus*, some representative DEGs were categorized into eight groups: glycolytic pathway, aflatoxin biosynthesis, ergosterol biosynthesis, cell wall, ribosome, response to oxidative stress, carbonyl stress response/carbohydrate metabolism, and signal transduction/stress-responsive translation/apoptosis regulation (Table 2). The gene *pfkA*, a key rate-limiting enzyme in the glycolytic pathway. Based on the *p*-value and rich factor, the aflatoxin biosynthesis pathway and steroid biosynthesis pathway were identified as target pathways (Figures 6c–e). These pathways included four differentially expressed genes, such as *aflD*, *adhA*, *erg5* and *dhcr7*. *aflD* was up-regulated gene, *adhA*, *erg5* and *dhcr7* were down-regulated genes. Additionally, cell wall related genes *Crf1*, *MNN2*, and *amyA*, and antioxidant-related gene *Lot6* and *catB* were significantly down-regulated.

**TABLE 2:** Classification of representative DEGs in glycolytic pathway, aflatoxin biosynthesis, ergosterol biosynthesis, cell wall, ribosome and response to oxidative stress.

Gene ID	Log_2_FC	Style	Gene Name	Description
Glycolytic pathway				
F9C07_4614	1.14	Up	*Aldh*	Aldehyde dehydrogenase (NAD+)
F9C07_1374	1.39	Up	*akr1A1*	Alcohol dehydrogenase (NADP+)
F9C07_2173698	-1.48	Down	*pfkA*	6-Phosphofructo kinase
Aflatoxin biosynthesis				
F9C07_5016	1.26	Up	*aflD*	Norsolorinic acid ketoreductase
F9C07_2282819	-1.23	Down	*adhA*	5’-Hydroxyaverantin dehydrogenase
Ergosterol biosynthesis				
F9C07_2128664	-1.56	Down	*erg5*	Sterol 22-desaturase
Cell wall				
F9C07_9102	-1.04	Down	*MNN2*	Alpha 1,2-mannosyltransferase
F9C07_1116	-1.20	Down	*Crf1*	Extracellular cell wall glucanase
F9C07_1989	-1.60	Down	*amyA*	Alpha-amylase
F9C07_2286761	-2.03	Down	*RodA*	Peptidoglycan polymerase
Ribosome				
F9C07_2279867	1.04	Up	RP-S3Ae	Small subunit ribosomal protein S3Ae
F9C07_7178	1.12	Up	*RP-L34e*	Large subunit ribosomal protein L34e
F9C07_1615	1.13	Up	*RP-L9e*	Large subunit ribosomal protein L9e
F9C07_2227618	1.16	Up	*RP-L22e*	Large subunit ribosomal protein L22e
F9C07_2279882	1.18	Up	*RP-L23Ae*	Large subunit ribosomal protein L23Ae
F9C07_8342	1.22	Up	*RP-L35e*	Large subunit ribosomal protein L35e
F9C07_2281179	1.28	Up	*RP-L18e*	Large subunit ribosomal protein L18e
F9C07_5779	1.28	Up	*RP-LP2*	Large subunit ribosomal protein LP2
F9C07_11086	1.37	Up	*RP-S3e*	Small subunit ribosomal protein S3e
F9C07_2287060	1.43	Up	*RP-S2e*	Small subunit ribosomal protein S2e
F9C07_2280103	1.67	Up	*RP-S12e*	Small subunit ribosomal protein S12e
Oxidative stress				
F9C07_1299813	1.18	Up	*CYB5R*	Cytochrome-b5 reductase
F9C07_8414	1.38	Up	*PDR*	ATP-binding cassette
F9C07_2850	-1.06	Down	*Lot6*	Putative NADPH-dependent FMN reductase
F9C07_3338	-1.11	Down	*CDC28*	Cyclin-dependent serine/threonine-protein kinase
F9C07_2285981	-1.11	Down	*SOD1*	Superoxide dismutase
F9C07_2195	-1.21	Down	*SIR2*	NAD-dependent protein deacetylase
F9C07_2285120	-1.24	Down	*catB*	Catalase
F9C07_2203617	-1.29	Down	*CHEK2*	Checkpoint kinase 2
Carbonyl stress response/carbohydrate metabolism				
F9C07_2282956	1.12	Up	*GLX3*	D-lactate dehydratase
Signal transduction/Stress-responsive translation/Apoptosis regulation				
F9C07_11189	1.18	Up	*rack1*	RACK1 scaffold protein

### 3.7 Effect of crude extract on AFB1 production and gene expression in AFB1 biosynthesis

**FIGURE 7:**
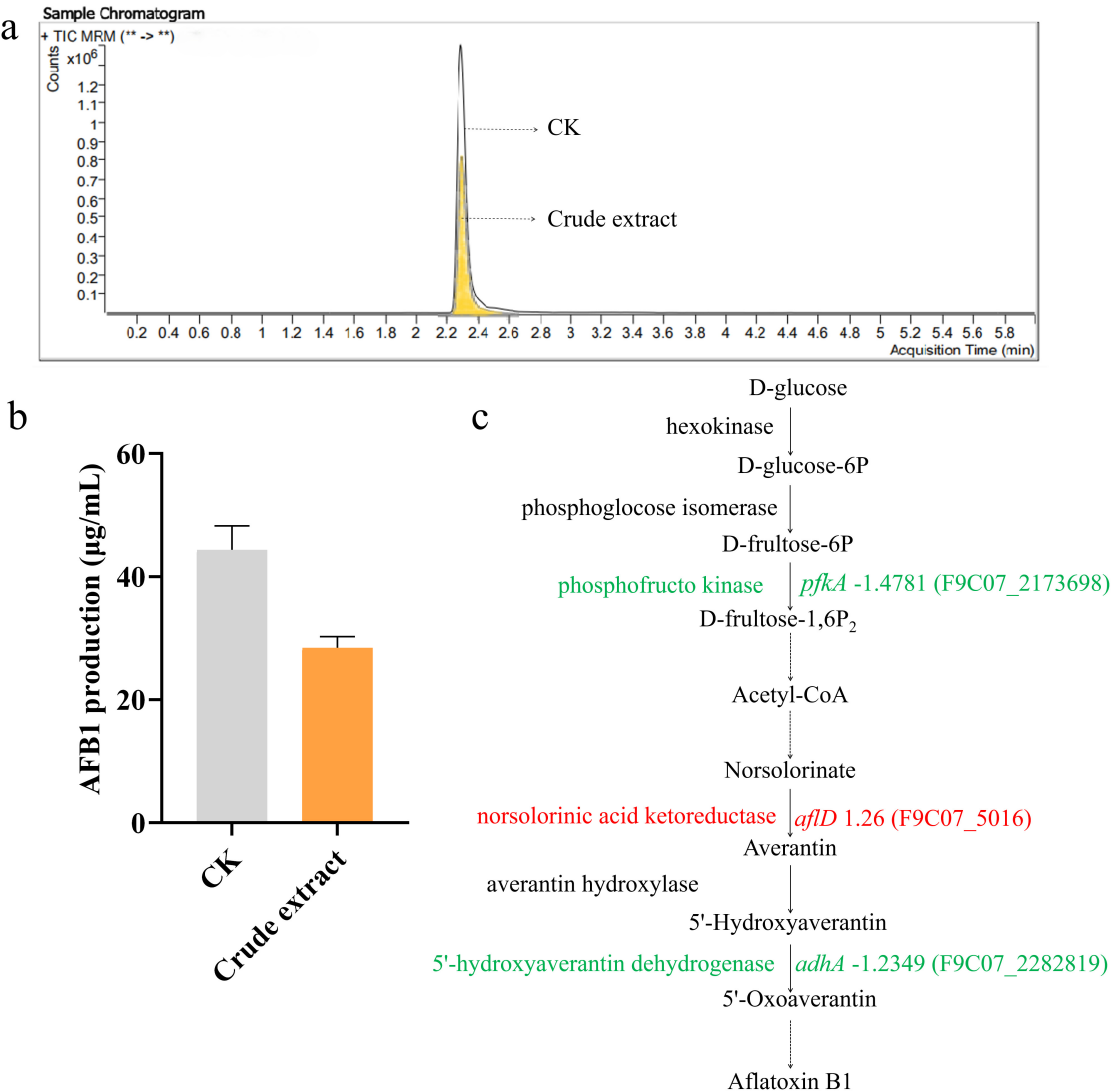
Effect of crude extract on AFB1 production and expression of genes involved in AFB1 biosynthetic pathway. **(a)** LC-MS analysis. **(b)** AFB1 production in PDB medium after 5 d. **(c)** Transcriptomic analysis of genes involved in the AFB1 biosynthetic pathway.

To investigate the effect of crude extract on AFB1 production, AFB1 levels in both treated and control samples were quantified using LC-MS. The DEGs involved in AFB1 biosynthetic pathway were also analyzed. The results showed that AFB1 production was significantly inhibited by the crude extract (Figures 7a,b). The AFB1 concentration in the treated group was 28.43 ± 1.84 μg/mL, representing a 35.88% decrease, compared to the control (44.34 ± 3.91 μg/mL) (Figure 7b). Within the AFB1 biosynthetic pathway (Figure 7c), we focused on the genes *pfkA*, *aflD*, and *adhA*, which encode phosphofructo kinase, norsolorinic acid ketoreductase, and 5’-hydroxyaverantin dehydrogenase, respectively. Although *aflD* was up-regulated 1.3-fold, *pfkA* and *adhA* were down-regulated 1.5- and 1.2-fold, respectively. These results suggest that the crude extract of *N. chlamydospora* B143 can inhibit the biosynthesis of AFB1.

### 3.8 Effect of crude extract on the cell membrane and MMP of *A. flavus*

**FIGURE 8:**
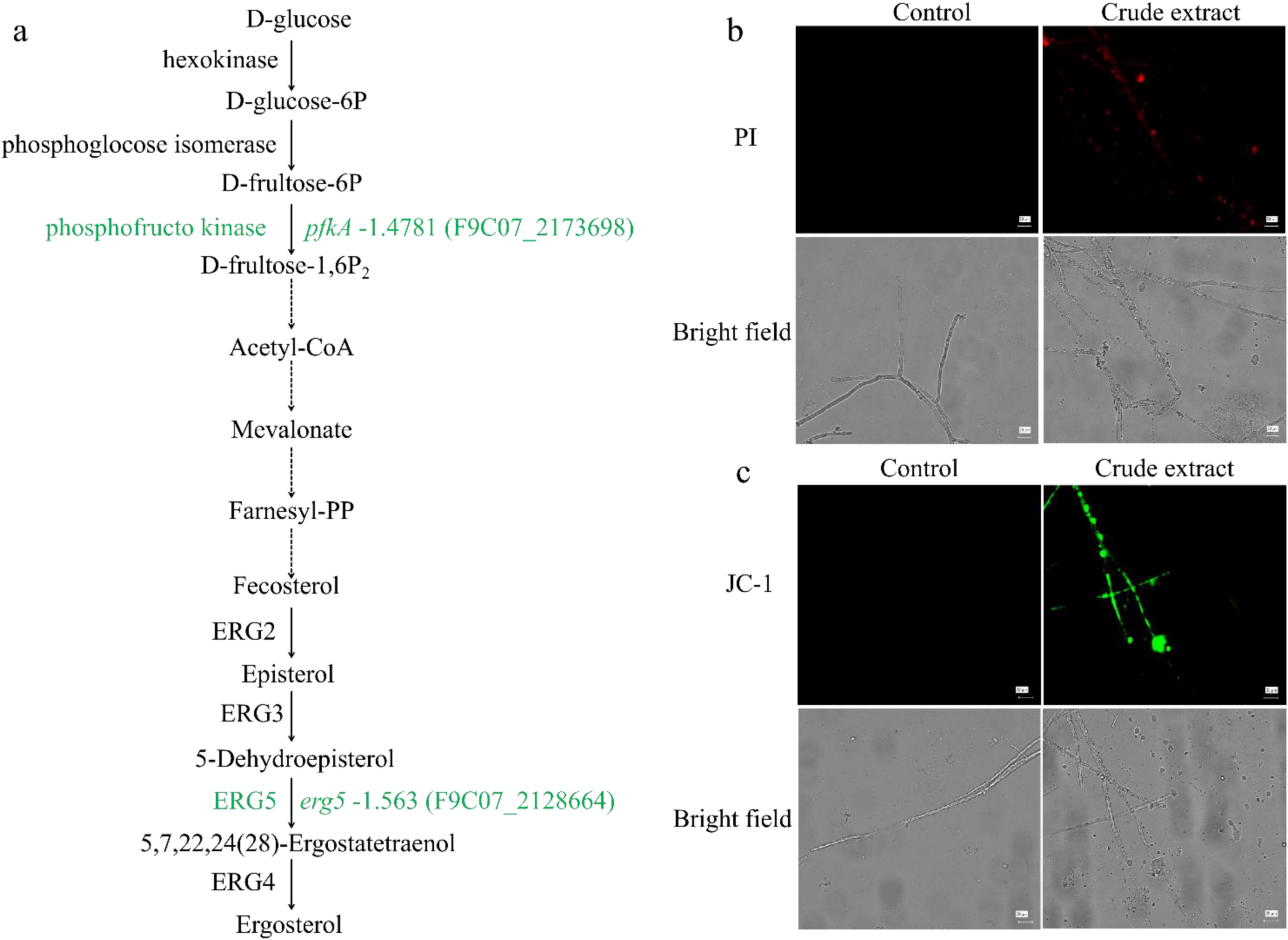
Effect of crude extract on cell membrane of *A. flavus*. **(a)** Transcriptomic analysis of genes involved in the ergosterol biosynthetic pathway. **(b)** Cell membrane integrity of *A. flavus* stained with PI after 72 h of incubation with crude extract. **(c)** MMP analysis of *A. flavus* treated with crude extract.

According to transcriptome data, genes erg5 and dhcr7, which encode sterol 22-desaturase and 7-dehydrocholesterol reductase in steroid biosynthesis pathway, were down-regulated. Among these, *erg5* was down-regulated 1.6-fold in the ergosterol biosynthesis pathway (Figure 8a). To validate the transcriptome results regarding the effect of crude extract from *N. chlamydospora* B143 on *A. flavus*, we performed PI and JC-1 staining to evaluate cell membrane integrity and changes in MMP, respectively. No red fluorescence was observed in the control group, whereas the mycelia treated with crude extract exhibited strong red fluorescence (Figure 8b). JC-1 staining results revealed no green fluorescence in the control group, while the treatment group displayed distinct and bright green fluorescence (Figure 8c). These results indicate that the crude extract of *N. chlamydospora* B143 damages cell membrane integrity and permeability of *A. flavus*, leading to cell apoptosis and ultimately inhibiting mycelial growth.

## 4 Discussion

Currently, 46 species have been reported in genus of *Nigrograna*, isolated from diverse substrates including soil, branches, pollen, stems, wood, and human foot (de Gruyter et al., 2013; Zhao et al., 2018; Mapook et al., 2020; Li et al., 2023; Zhang et al., 2024; Rakshit and Sarma, 2025). Among these, 25 species have been recorded from China. In this study, a new species, *N. chlamydospora* sp. nov., was isolated from marine sediment in Guangxi province, China. This is the first report of *Nigrograna* species from the marine environment. Only the asexual morph was observed, characterized by the presence of oval to ellipsoid chlamydospores, conidia were not detected. Phylogenetic analysis showed that strains B141, B143, B373, B375, and B393 clustered within two subclades together with *Nigrograna rhizophorae*. However, *N. rhizophorae* differs from *N. chlamydospora* in producing ellipsoidal to cylindrical conidia measuring 3–4.5 × 1–2 μm (Dayarathne et al., 2020). Additionally, we found it particularly challenging to induce conidial production in *N. chlamydospora* under laboratory conditions.

This is the first report of antifungal activity against *A. flavus* by any species within the genus *Nigrograna*. Specifically, *N. chlamydospora* B143 showed an inhibition rate of 44.16 ± 6.14% against *A. flavus*, a level of activity comparable to that reported for other microbial antagonists. For instance, *Alternaria alstroemeriae* F1 and *Alternaria burnsii* F2 achieved inhibition rates of 54.91 ± 0.55% and 44.95 ± 1.19%, respectively (Ye et al., 2025). While the bacterial strains *Bacillus siamensis* 3BS12-4 and *Pseudomonas palleroniana* B-BH16-1 showed 43.1 and 40.1% inhibition, respectively (Aphaiso et al., 2024; Zhou et al., 2023). Notably, *Auricularia auricula* H29 reached 59.6% inhibition, albeit after an extended 216 h of interaction period (Yang et al., 2024). Several factors may account for the observed variations in antifungal potency among these studies. First, differences in the source organisms and their ecological niches likely influence the repertoire of secondary metabolites produced. As a marine-derived fungus, *N. chlamydospora* B143 may produce distinct bioactive compounds compared to terrestrial endophytic fungi or bacteria, potentially explaining why its activity falls within the mid-range of reported values despite belonging to a previously unexplored genus. Second, experimental conditions such as co-culture duration, medium composition, and assay protocols can substantially affect the measured inhibition rates—exemplified by the 59.6% inhibition reported for *A. auricula*, which required 216 h of interaction compared to our standard 5-day assay. The fact that *N. chlamydospora* B143 exhibits activity comparable to known biocontrol agents from well-studied genera (e.g., *Alternaria*, *Bacillus*, *Pseudomonas*) is particularly noteworthy. This suggests that marine-derived *Nigrograna* species represent an underexplored reservoir of antifungal compounds worthy of further investigation. Moreover, as this is the first report of antifungal activity within the genus, our findings establish a foundation for future chemical and mechanistic studies.

Aphaiso et al. (2024) reported similar results, showing that extracellular compounds from *Bacillus siamensis* 3BS12-4 inhibited the growth of *A. flavus*. Similarly, an ordinary extract of *Agaricus bisporus* was found to suppress *A. flavus* growth, albeit with a lower inhibition rate of 43.95% (Hussein and Owied, 2024). The highest inhibition rate against *A. flavus* reported was observed with VOCs produced by *Candida nivariensis* DMKU-CE18, reaching 64.9 ± 7.0% (Jaibangyang et al., 2020). These findings are consistent with the results of our study. Previous reports have shown that microbial bioactive compounds can inhibit fungal growth by inducing morphological abnormalities in hyphae. For instance, Aphaiso et al. (2024) observed malformed, folded, and dented mycelia in *A. flavus* after treatment with extracellular compounds from *Bacillus siamensis* 3BS12-4. Similarly, Yang et al. (2019) reported that the VOCs from *Streptomyces alboflavus* TD-1 caused severe wrinkling and irregular distortions of hyphae.

To the best of our knowledge, the inhibition rates of hyphal growth and spore germination (70.84 ± 4.29% and 98.23 ± 0.10%) observed in this study are among the highest reported to date. For instance, a 25% cell-free culture filtrate of *Bacillus subtilis* B-FS06 reduced the spore germination rate of *A. flavus* by only 40% (Zhang et al., 2008). Treatment with the cell-free fermentation supernatant of *Bacillus velezensis* 906 resulted in a spore germination rate of approximately 20% (Li et al., 2024). Jaibangyang et al. (2020) reported that 46 yeast strains inhibited the conidial germination of *A. flavus* within a range of 9.3 ± 13.6% to 49.3 ± 8.7%. The pronounced suppression of spore germination demonstrated in this work highlights the potential of the crude extract from *N. chlamydospora* B143 as an effective antifungal agent.

While the petroleum ether crude extract of *N. chlamydospora* B143 demonstrated significant antifungal activity at 25 mg/mL, it is important to consider the practical feasibility of this concentration for real-world applications. As a crude extract, this concentration reflects the combined mass of all extracted compounds, with the active constituents likely representing only a minor fraction. The effective concentration of the purified active compound(s) is therefore expected to be substantially lower. Future studies should focus on bioactivity-guided isolation of the specific antifungal metabolites, followed by structural characterization and optimization. Once identified, these compounds could be evaluated in formulation studies aimed at enhancing stability and bioavailability. Potential application scenarios, such as seed coating or controlled-atmosphere storage, may also allow for effective use of the extract at practical concentrations, particularly when integrated with other preservation methods. Ultimately, field trials and cost-benefit analyses will be necessary to determine the economic viability of this approach.

It has been reported that the transcriptome of *A. flavus* is readily influenced by microbial active substances (Maud et al., 2023; Zhang et al., 2025), which is consistent with our research findings. Ye et al. (2025) reported 122 DEGs, including 86 upregulated and 36 downregulated genes in *A. flavus* when inhibited by *A. alstroemeriae* F1 compared to an untreated control. These DEGs were involved in metabolic pathways, biosynthesis of amino acids, biosynthesis of secondary metabolisms, among others.

Transcriptomic analysis revealed that *adhA* was down-regulated, while *aflD* was up-regulated. This pattern differs from previous reports. AFB1 biosynthesis is a highly complex process regulated by a large gene cluster comprising approximately 30 genes, such as *aflA*/*fas-2*, *aflB*/*fas-1*, *aflC*/*pksA*, *aflD*/*nor-1*, *aflE*/*norA*, *aflF*/*norB*, *aflG*/*avnA*, and *aflH*/*adhA* (Bhatnagar et al., 2006). Down-regulation or destruction of any of these genes can influence AFB1 production. For example, *Alternaria alstroemeriae* F1 was found to reduce AFB1 by down-regulating 14 genes and up-regulating 2 genes (Ye et al., 2025). The volatile compound 2-ethylhexanol of microbial or plant origin down-regulated *aflD*, *laeA*, *velB*, and *veA*, while up-regulated *aflM*, *aflP*, and *aflR* (Zhang et al., 2025). Similarly, VOCs from Streptomyces alboflavus TD-1 decreased AFB1 production by down-regulating 8 genes, including *laeA*, *veA*, *aflR*, *aflS*, *aflD*, *aflM*, *aflP*, and *aflQ* (Yang et al., 2019). Estragole from *Pimenta racemosa* affected the expression of 6 genes in the AFB1 biosynthetic pathway, down-regulating *aflO*, *aflQ*, *aflU*, *aflT*, *aflB*, and *aflC* (Liang et al., 2023).

An intriguing finding from the transcriptomic analysis was the divergent regulation of genes within the aflatoxin biosynthesis cluster: the early pathway gene *aflD* (encoding norsolorinic acid ketoreductase) was significantly upregulated, while the late pathway gene *adhA* (encoding 5’-hydroxyaverantin dehydrogenase) was markedly downregulated, concomitant with a substantial reduction in AFB1 production. This apparent contradiction may be explained by several non-mutually exclusive mechanisms. Firstly, aflatoxin biosynthesis is a multi-step pathway in which the final output is determined by the rate-limiting step, rather than the sum of all transcriptional activities. Even if early steps are transcriptionally enhanced, a severe blockade at a later step—such as that catalyzed by *adhA*—could create a metabolic bottleneck, preventing the completion of the pathway and resulting in reduced final toxin yield. This “bottleneck hypothesis” is consistent with the central role of *adhA* in the later stages of aflatoxin assembly. Secondly, the upregulation of *aflD* may represent a compensatory feedback response. The fungus might sense an interruption in downstream flux (e.g., accumulation of intermediates due to *adhA* inhibition) and attempt to overcome this by increasing the expression of upstream genes. However, if the downstream block is insurmountable, this compensatory effort would fail to restore toxin production. Thirdly, it is important that transcript levels do not always correlate with protein abundance or enzymatic activity. Post-transcriptional regulation, translational efficiency, or protein degradation could modulate the functional output of *aflD*, potentially nullifying the effect of its increased mRNA expression. Finally, the possibility of metabolic shunting cannot be excluded. Intermediates from the aflatoxin pathway might be diverted toward other secondary metabolites (e.g., antioxidant compounds) under stress conditions induced by the extract, thereby reducing the carbon flux available for AFB1 synthesis. Taken together, while the precise mechanism underlying this transcriptional divergence requires further investigation, the consistent downregulation of *adhA* across multiple replicates, coupled with its well-established role in aflatoxin biosynthesis, supports our conclusion that interference with this late-stage gene contributes significantly to the observed reduction in AFB1 production. Future studies employing protein-level analysis (e.g., western blotting or enzymatic activity assays) and metabolite profiling of pathway intermediates would help to distinguish between these possibilities and definitively establish the functional consequences of the observed transcriptional changes.

Ergosterol is a major component of the fungal cell membrane and plays a significant role in ensuring membrane integrity, membrane fluidity, membrane stability, cell viability, the activity of membrane-bound enzymes, cellular transport processes, and other functions (Dupont et al., 2012; Krumpe et al., 2012). The biosynthesis of ergosterol is a highly complex and energy-intensive process involving approximately 22 genes. The pathway from acetyl-CoA to ergosterol can be divided into three stages: Mevalonic acid (MVA), farnesyl pyrophosphate (FPP), and the ergosterol synthesis pathway (Hu et al., 2017). The ergosterol synthesis pathway is the most complex and critical stage, involving 13 genes including *erg9*, *erg1*, *erg7*, *erg11*, *erg24*, *erg25*, *erg26*, *erg27*, *erg6*, *erg2*, *erg3*, *erg4*, and *erg5* (Klug and Daum, 2014). The gene *erg5* encodes C-22 sterol dehydrogenase, which catalyzes the conversion of ergosta-5,27,24(28)-trienol to ergosta-5,7,22,24(28)-tetraenol. In this study, the crude extract treatment was found to damage cell membrane integrity of *A. flavus*. Transcriptomic analysis showed that only *erg5* was down-regulated in the ergosterol synthesis pathway, rather than multiple genes, and this finding was consistent with the PI staining results. However, *erg5* has been reported as a non-essential gene in previous studies (Hu et al., 2017), which contrasts with our results. For example, 2-ethylhexanol was shown to reduce ergosterol content by down-regulating *erg1*, *erg6* and *erg24* (Zhang et al., 2025). Additionally, significant down-regulation of ergosterol-related genes such as *erg9*, *erg1*, *erg7*, *erg11*, *erg24*, *erg25*, *erg26*, *erg27*, and *erg4* had been observed in other studies (Xu et al., 2021). These findings indicate that the crude extract of *N. chlamydospora* B143 inhibits the growth of *A. flavus* by disrupting the structure of cell membrane and suppressing ergosterol synthesis. Therefore, transcriptomic analysis revealed that the crude extract inhibits the mycelial growth and AFB1 production by two different pathways.

The severe morphological disruption, membrane permeabilization, and loss of mitochondrial membrane potential observed upon treatment with the crude extract point to the plasma membrane and associated organelles. While transcriptomic analysis revealed downregulation of *erg5*, a gene involved in late stages of ergosterol biosynthesis, this single transcriptional change is unlikely to account for the dramatic phenotypes observed, particularly given that *erg5* is considered a non-essential gene in fungi. We now propose the active compounds in the crude extract likely insert into the lipid bilayer, directly disrupting membrane integrity and mitochondrial membrane potential, which as direct membrane interaction. This physical disruption is the primary cause of the observed lethality. The down-regulation of *erg5* may be a consequence of the cellular stress response or an additional, but not primary, factor contributing to the overall phenotype, representing a secondary effect. Distinguishing between direct membrane disruption and inhibition of ergosterol biosynthesis as the primary mechanism would require targeted biochemical approaches, such as membrane fluidity measurements using fluorescence polarization, quantification of ergosterol content by HPLC, or direct assessment of compound-membrane interactions using model lipid bilayers. Nevertheless, the present data strongly support the membrane as a key target, with direct physical interaction representing the most likely primary mode of action.

## 5 Conclusion

**FIGURE 9:**
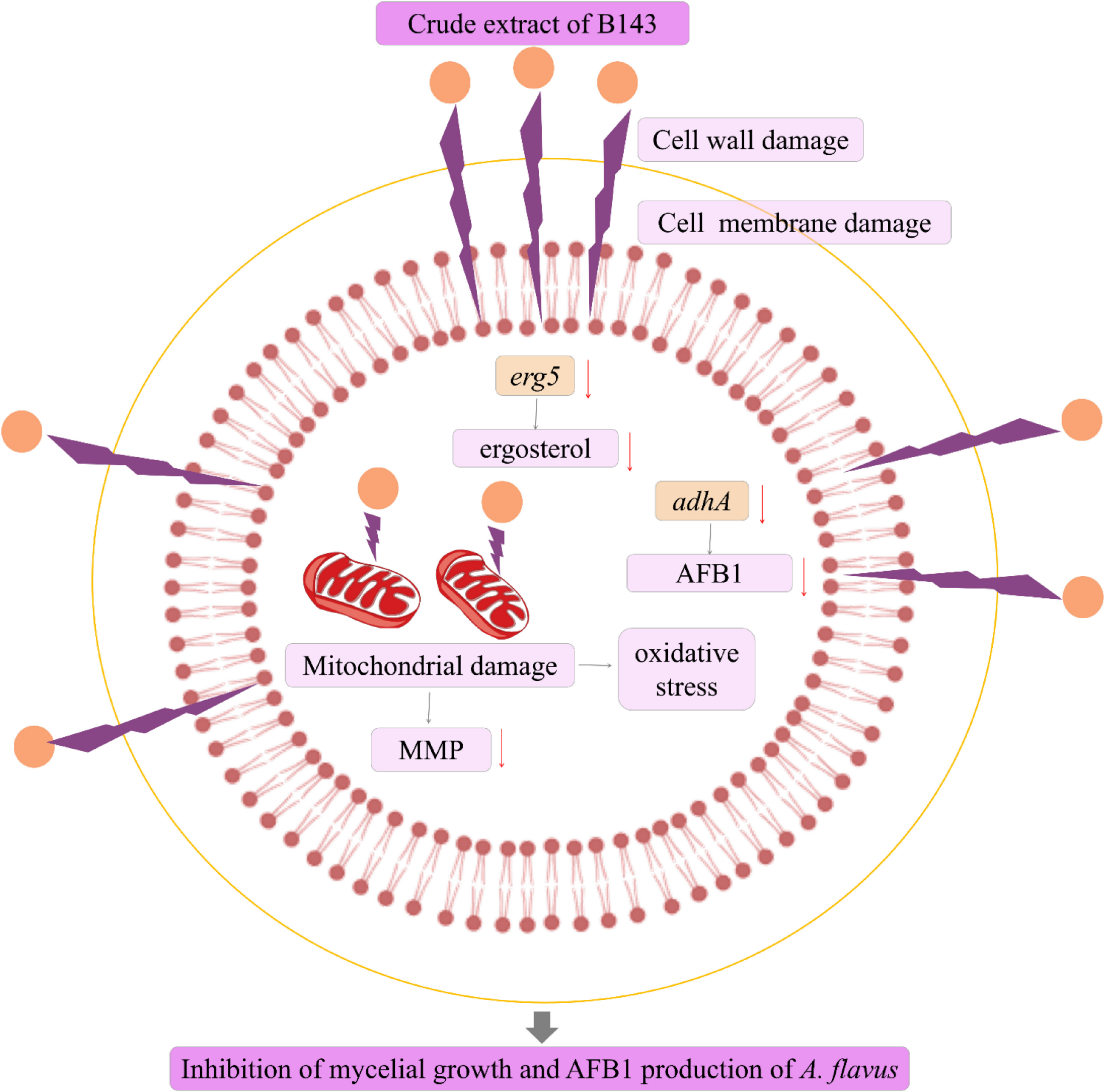
A schematic diagram illustrating the mechanism of action of the crude extract from *N. chlamydospora* B143 against *A. flavus*.

In this study, we isolated and identified a new species *N. chlamydospora* sp. nov., based on morphological and molecular characteristics. This species demonstrated significant inhibitory effects on the mycelial growth of *A. flavus*. Furthermore, the crude extract derived from *N. chlamydospora* B143 strongly suppressed both vegetative growth and spore germination of *A. flavus* by adversely affecting hyphal morphology and structure integrity. Additionally, the crude extract also inhibited AFB1 production. To elucidate the underlying mechanisms, we conducted transcriptomic analysis followed by validation experiments to explore how the crude extract impedes mycelial growth and AFB1 biosynthesis in *A. flavus* (Figure 9). Transcriptomic profiling revealed that treatment with crude extract led to a marked reduction in ergosterol content and AFB1 production. This effect was achieved through the down-regulation of key genes involved in the ergosterol biosynthesis pathway (e.g., *erg5*) and the AFB1 biosynthesis pathway (e.g., *adhA*). Moreover, the crude extract disrupted cell wall and cell membrane integrity and induced oxidative stress, further contributing to the inhibition of fungal growth. The novelty of this study lies in the first report of the new species *Nigrograna chlamydospora* sp. nov. as a dual-action antagonist against *A. flavus*, and in identifying genes *erg5* and *adhA* as potential targets for inhibiting both mycelial growth and AFB1 production. In conclusion, *N. chlamydospora* B143 exhibits great potential as a promising biological control agent for effectively mitigating *A. flavus* contamination in grains and agricultural products. It is worth noting that the search of antifungal agents, and future work will focus on bioassay-guided fractionation to isolate and identify the specific secondary metabolites responsible for the observed inhibition of fungal growth and aflatoxin biosynthesis.

## Data Availability

The datasets presented in this study can be found in online repositories. The names of the repository/repositories and accession number(s) can be found at: https://www.ncbi.nlm.nih.gov/genbank/, PV 839861, PV 839905, PV 839924, PX278198, https://www.ncbi.nlm.nih.gov/genbank/, PV 839862, PV 839906, PV 839925, PX278199, https://www.ncbi.nlm.nih.gov/genbank/, PV 839863, PV 839907, PV 839926, PX278200, https://www.ncbi.nlm.nih.gov/genbank/, PV 839864, PV 839908, PV 839927, PX278201, https://www.ncbi.nlm.nih.gov/genbank/, PV 839865, PV 839909, PV 839928, PX278197.

## Footnotes

1
http://www.ncbi.nlm.nih.gov

2
http://www.ncbi.nlm.nih.gov/genbank/
